# Commentary: Meta-analysis of the effect and clinical significance of Delphian lymph node metastasis in papillary thyroid cancer

**DOI:** 10.3389/fendo.2024.1392174

**Published:** 2024-09-26

**Authors:** Shanshan Wu

**Affiliations:** Clinical Laboratory Department, The Affiliated Hospital of Liaoning University of Traditional Chinese Medicine, Shenyang, Liaoning, China

**Keywords:** papillary thyroid carcinoma, Delphian lymph node, lymph node metastases, thyroid cancer, extrathyroidal extension

I would like to share my views on the recent article titled “Meta-analysis of the effect and clinical significance of Delphian lymph node metastasis in papillary thyroid cancer”, authored by Chen and colleagues (1). During my reading, I noticed certain data entry errors in Chen’s analysis, which consequently impacted the results of the corresponding analyses. The analysis results after data correction showed that the rate of Delphian lymph node (DLN) positivity among male papillary thyroid carcinoma (PTC) patients was 2.08 times higher than that of DLN-negative cases. But in contrast, no significant difference was observed between male and female PTC patients in the rate of DLN positivity in Chen’s study. Additionally, I have identified several issues with the forest plots presented in Chen‘s research.

In order to prevent misleading more readers, we suggest the following modifications in line with a rigorous scientific attitude:

1. In Figure 2C (1), the total cases is 46 in the DLN-Positive group of included study Chai 2013 (2), instead of 283.

2. In Figure 3B (1), the study of Zuo 2022 should have 9 cases lateral lymph node metastasis (LLNM) in the DLN-negativity group instead of 106 (3).

3. In Table 3 (1), the number of DLN-Positive cases in the data column for Oh 2013 should be 49, and the number of DLN-Negative cases should be 196, making the total sample size for this study is 245 (4). For Zuo 2022, the number of DLN-Positive cases should be 106, the number of DLN-Negative cases should be 416, resulting in a total sample size of 522 (3). Across the five studies included, the number of DLN-Positive cases should be 716, the number of DLN-Negative cases should be 2512, with a total sample size of 3228.

4. There are still some formatting errors in Figures 2B, D–G forest plots (1). According to the data in the data column, it can be inferred that the forest plots should have “DLN-Positive” on the left and “DLN-Negative” on the right of the invalid line.

5. There are also some formatting errors in the Figures 3A-C forest plots (1). According to the data column, the left side of the invalid line should be labeled “DLN-Positive” and the right side should be labeled “DLN-Negative”.

6. The results section contains a repetition of content between “3.2.5 ETE” and “3.2.4 Multifocality”on page four, line 13-22. It is suggested to modify “3.2.5 ETE” to the following: The results showed that the rate of DLN-Positive in patients with PTC having extrathyroidal extension (ETE) of the thyroid was 2.40 times higher than that of DLN-negative (OR=2.40, 95% CI: 1.95-2.96, P < 0.00001, Figure 2H) (1). It can be inferred that patients with PTC having DLNM are at a higher risk of ETE.

## Statistical analysis

All data were analyzed using RevMan version 5.3 and SPSS 27.0 software. The odds ratio (OR) with 95% confidence interval (CI) was used for dichotomous variables. The I² value indicated the heterogeneity among included original studies; I² values of over 25%, 50%, and 75% are commonly defined as low, medium, and high heterogeneity, respectively. When I² ≥ 50%, the heterogeneity is significant, this degree of variability required sensitivity analysis or subgroup analysis to identify plausible sources of heterogeneity,and the random effect model is applied. When I² < 50%, the heterogeneity among included studies is considered small, and the fixed effects model is selected. A *P* value of < 0.05 was considered statistically significant.

## Revised meta-analysis results

**Figure 1:**
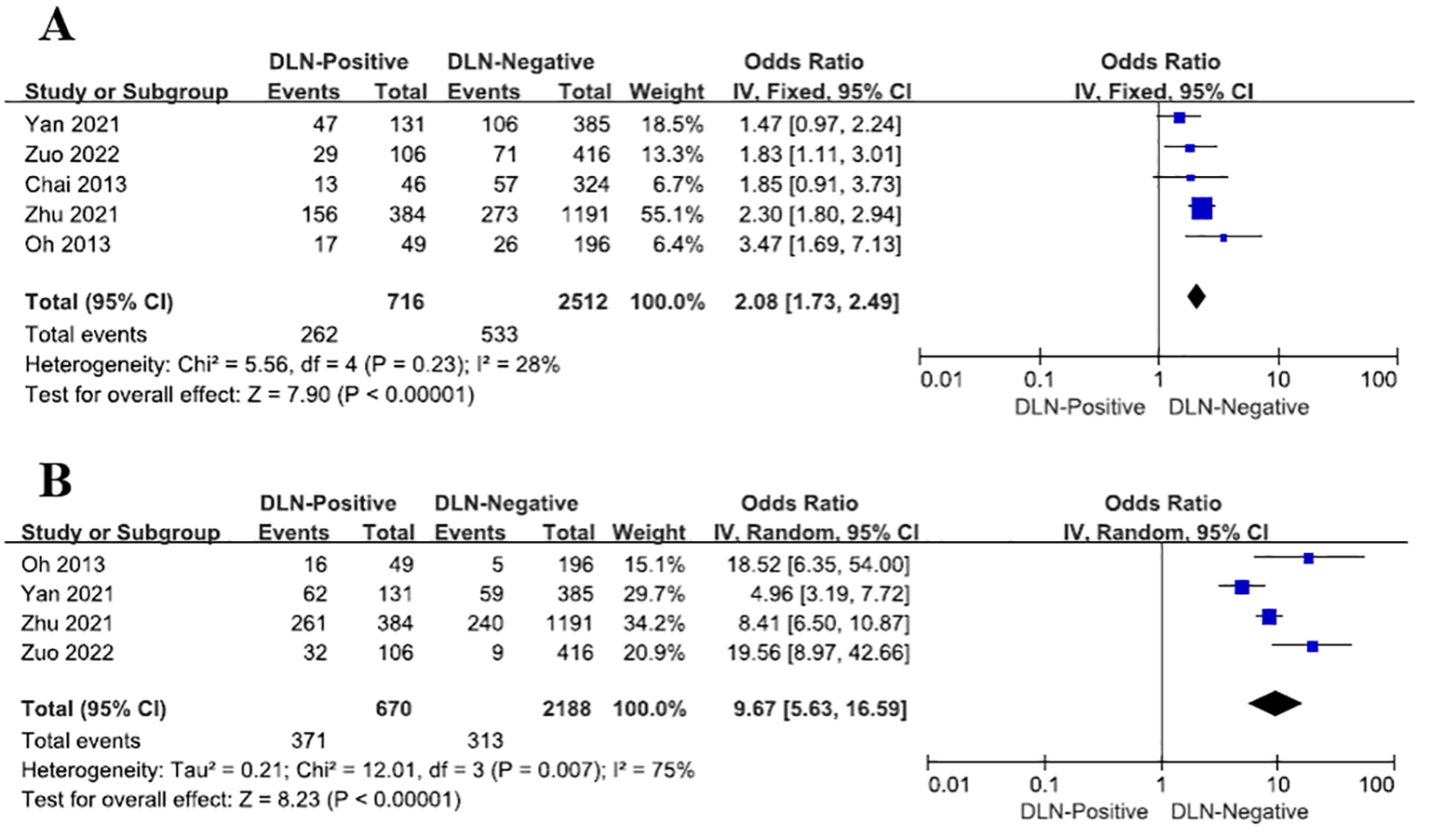
Characteristics with regard to male **(A)**, to other compartments regarding LLNM **(B)**.

1. The result of the reanalysis revealed that the rate of DLN positivity among male papillary thyroid carcinoma (PTC) patients was 2.08 times higher than DLN-negative cases (OR = 2.08, 95% CI: 1.73–2.49, P<0.00001, **Figure 1A**), after data correction, the result has statistical significance. In summary, male PTC patients are more likely to experience Delphian lymph node metastasis (DLNM).

2. Upon reanalysis, DLN-Positive patients exhibited a 9.67-fold higher risk of LLNM compared to DLN-Negative cases (OR = 9.67, 95% CI: 5.63–16.59, P<0.00001, **Figure 1B**). After adjustment, the inter-study heterogeneity (I^2^) among the studies decreased from 94% to 75%.

3. DLN was classified into negative and positive groups based on detection, in order to compare the differences in DLN metastasis rates across the included studies. Upon reanalysis, the results revealed P < 0.05, indicating unequal DLN metastasis rates across the five included papers, the statistical significance of the corrected results remained unchanged. (**Supplementary Table 1**).

Gender disparities in the incidence and prognosis of various malignancies, including thyroid cancer, underscore the need for tailored management strategies (5–7). The incidence of PTC in females is approximately threefold higher than in males (8), but the mortality rate in females is only 1.3 times higher than in males (9), indicating that male sex is associated with aggressive behavior and poor prognosis. The result of our reanalyzed the rate of DLN positivity was 2.08 times higher than that of DLN-negative, and that DLN-Positive patients are more likely to experience CLNM, LLNM, and ETE, supports this observation. A survey conducted on a Chinese PTC population showed that the incidence of non-microcarcinoma (nM-PTC), CLNM, LLNM, advanced disease, and bilateral disease is higher in males than in females. However, the proportion of males with Hashimoto’s thyroiditis (HT) is significantly lower than that of females. Moreover, moderate-risk and high-risk factors have a higher incidence in male PTC patients (10). Therefore, male DLN-Positive PTC patients should receive increased attention. When DLN metastasis is not detected in papillary thyroid microcarcinomas (PTMC), ipsilateral thyroid lobectomy and central neck lymph node dissection should be performed. Even if preoperative examinations do not detect lateral neck LNM, clinicians should remain vigilant for such involvement during follow-up if DLN metastasis is discovered postoperatively (11). Meta-analysis conducted by Bin Wang et al. concluded that DLNM in PTC patients is strongly correlated with multifocality, bilaterality, ETE, lymphovascular invasion, further CLNM, and LLNM (12). The above conclusions were similarly validated in the study by Chen et al. DLNM in PTC patients is associated with many adverse prognostic factors. Additionally, DLNM indicates a higher risk of CLNM in patients and a greater likelihood of further LLNM occurrence. It is recommended to assess DLN in all thyroid cancer patients and consider whether dissection is necessary based on individual circumstances. If there is suspicion of DLNM, surgeons should meticulously dissect the central neck compartment and give special consideration to the lateral lymph node compartments (4).

In conclusion, it is wise to develop a personalized treatment plan based on the specific condition of the patient.
