# Supplementary material for: Commentary: Meta-analysis of the effect and clinical significance of Delphian lymph node metastasis in papillary thyroid cancer
Source: Front Endocrinol (Lausanne). 2024 Sep 26;15:1392174. doi: 10.3389/fendo.2024.1392174 (PMC11464285; doi:10.3389/fendo.2024.1392174)
Supplement: Supplementary file 1 [file DataSheet1.pdf]

SUPPLEMENTARY TABLE 1. Basic information and chi-square tests of DLNM.

Study * DLN Crosstabulation				
Count				
		DLN		Total
		Positive	Negative	
Study	Chai 2013	46	324	370
	Oh 2013	49	196	245
	Yan 2021	131	385	516
	Zhu 2021	384	1191	1575
	Zuo 2022	106	416	522
Total		716	2512	3228

Chi-Square Tests			
	Value	df	symptotic Significance (2-sided)
Pearson Chi-Square	29.599[a]	4	.000
Likelihood Ratio	32.289	4	.000
Linear-by-Linear Association	10.880[b]	1	.001
N of Valid Cases	3228		

a. 0 cells (0.0%) have expected count less than 5. The minimum expected count is 54.34

b. The standardized statistic is -3.299
